# Infant feces-derived *Lactobacillus gasseri* FWJL-4 mitigates experimental necrotizing enterocolitis *via* acetate production

**DOI:** 10.1080/19490976.2024.2430541

**Published:** 2024-12-08

**Authors:** Li-Long Pan, Yudong Sun, Xiaoliang Dong, Zhengnan Ren, Binbin Li, Ping Yang, Le Zhang, Jia Sun

**Affiliations:** a Department of Neonatology, Affiliated Children’s Hospital of Jiangnan University (Wuxi Children’s Hospital), Wuxi School of Medicine, Jiangnan University, Wuxi, China; b School of Food Science and Technology, Jiangnan University, Wuxi, China

**Keywords:** Necrotizing enterocolitis, *Lactobacillus gasseri*, acetate, probiotics

## Abstract

Necrotizing enterocolitis (NEC) is a life-threatening disease in premature infants, characterized by high mortality. Recent studies increasingly highlight the role of gut dysbiosis in NEC pathogenesis. Although probiotics have shown some efficacy in preventing NEC, further research is needed to determine potential strains and approaches. In this study, we demonstrated that the novel probiotic strain *Lactobacillus gasseri* (*L. gasseri*) FWJL-4, isolated from the feces of healthy infants, significantly enhanced intestinal barrier function, providing substantial protection against NEC. This protective effect was attributed to elevated intestinal acetate levels. Notably, acetate supplementation alone was sufficient to mitigate NEC, mimicking the protective effects of *L. gasseri* FWJL-4. Mechanistically, we revealed that *L. gasseri* FWJL-4 inhibited necroptosis and preserved the number of the goblet cells and enterocytes through the production of the short-chain fatty acid acetate, *via* activation of the acetate receptors G protein-coupled receptor (GPR) 41 and GPR43. Our findings suggest that *L. gasseri* FWJL-4 enhances intestinal barrier function to protect against NEC, underscoring the potential of probiotic manipulation as a promising strategy for NEC prevention.

## GRAPHICAL ABSTRACT

**Figure:**
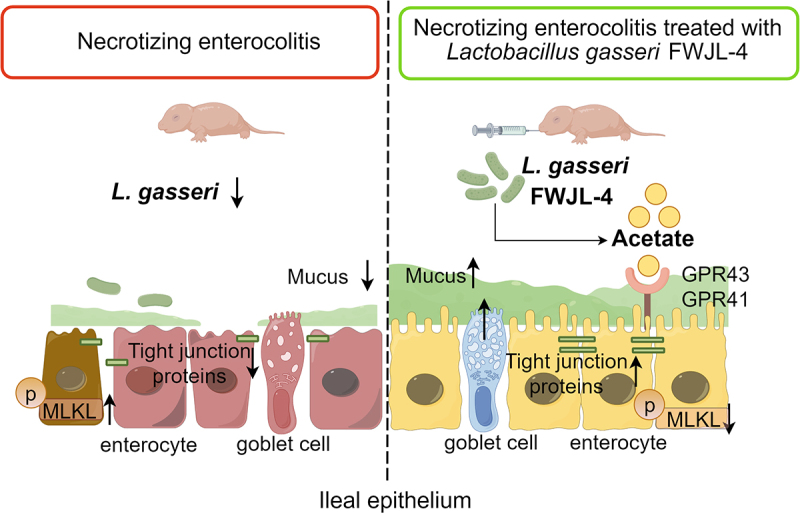
**A schematic diagram showing that *Lactobacillus gasseri (L. gasseri)* mitigates experimental necrotizing enterocolitis via acetate production.** During necrotizing enterocolitis (NEC), there is a decline in the population of protective bacteria, including *Lactobacillus gasseri (L. gasseri)*. Supplementation with a novel probiotic strain, *L. gasseri* FWJL-4, isolated from the feces of healthy infants, effectively alleviates experimental NEC through the production of acetate. Mechanistically, *L. gasseri* FWJL-4-derived acetate maintains the population of enterocytes and goblet cells while inhibiting ileal necroptosis via the GPR43 and GPR41 pathways. This action enhances gut barrier function and mitigates NEC in mice. The graphical abstract was created with FigDraw.com.

## Introduction

Necrotizing enterocolitis (NEC) is one of the most common and serious intestinal inflammatory diseases in neonates, primarily affecting premature infants. Characterized by localized necrosis of intestinal tissue caused by various factors, NEC has an average mortality rate ranging from 20% to 30%.^1^ The pathogenesis of NEC is multifactorial, primarily influenced by prematurity, gut dysbiosis, formula feeding and so on.^2^ The early postnatal period is a critical for the proper development and stabilization of the gut microbiome, making the maintenance of microbial homeostasis essential during this phase.^3^ Infants with NEC exhibit a distinct microbial composition compared to healthy counterparts, with an increased relative abundance of pathogenic bacteria being linked to a heightened risk of the disease.^4^ Disruption of the delicate microbial ecosystem in the gut plays a pivotal role in initiating and exacerbating the severity of NEC.^5^ This dysbiosis is often marked by a reduction in beneficial bacteria, such as *Bifidobacterium* and *Lactobacillus*, alongside an increase in harmful species like *Enterococcus faecalis*, *Klebsiella*, and *Clostridium perfringens*.^5–7^ Such an imbalance can compromise intestinal barrier integrity and intensify inflammatory response, thereby increasing the risk of NEC.

*Lactobacillus gasseri* (*L. gasseri*) is a natural resident of the human gastrointestinal tract and colonizes the intestine in the early weeks of life.^8^ As a commensal bacterium, *L. gasseri* plays a vital role in maintaining host intestinal homeostasis.^9^ For example, it produces bacteriocins that target intestinal epithelial cells, aiding in the resistance to diarrhea.^10^ Furthermore, *L. gasseri* demonstrates a range of positive effects on human health, encompassing anti-inflammatory, immune regulation, and antibacterial functions.^11^ Being a SCFAs-producing bacterium, *L. gasseri* can effectively generate SCFAs,^12^ thereby exerting various biological functions.^13^ However, clinical research has revealed a notable decrease in beneficial bacteria *L. gasseri* among children diagnosed with NEC.^7^ Simultaneously, microbiota-derived metabolites, including short-chain fatty acids (SCFAs), are markedly diminished during NEC.^14^ Furthermore, research has substantiated the positive impact of breast milk on neonatal development, emphasizing the crucial role of acetate a pivotal SCFA in early postnatal growth.^15^ Acetate is thought to exhibit anti-inflammatory properties and plays a key role in preserving the integrity of the intestinal mucosa.^16^ However, the role and mechanism of *L. gasseri* in NEC remain unclear.

Previously, we isolated an novel probiotic strain, *L. gasseri* FWJL-4, from the feces of healthy infants and demonstrated its excellent tolerance to gastrointestinal fluid and anti-inflammatory capacity *in vitro*.^11^ In this study, we conducted the first *in vivo* assessment of the protective role of *L. gasseri* FWJL-4 in NEC and elucidated the underlying mechanism.

## Methods

### Ethics

All experimental procedures involving mice were complied with the ARRIVE guidelines and carried out according to protocols approved by the Institutional Animal Ethics Committee of Jiangnan University (JN. No20230615c0300830[311]).

### Preparation of probiotic Lactobacillus gasseri FWJL-4

*L. gasseri* FWJL-4 was isolated from the feces of healthy infants, as previously reported,^11^ and is maintained in the in-house culture collection of the Guangdong Microbial Culture Collection Center (GDMCC No: 62365). The *Lactobacillus rhamnosus* GG (LGG, ATCC 53,103) obtained from American Type Culture Collection (ATCC) served as positive reference strain.^6,17^ The *Lactobacillus* strains were anaerobically cultured in deMan-Rogosa-Sharpe (MRS) medium at 37°C for 24 hours. Following incubation, cultures were plated on MRS agar at specific serial dilutions, and grown anaerobically at 37°C for 48–72 hours. For quantitative analysis of bacteria growth, a photometer was used to measure absorbance at 600 nm, comparing it against a standard curve of bacterial colony-forming units (CFU) per milliliter grown on MRS agar. Bacteria from the culture medium were harvested by centrifugation at 4,000 *g* for 15 minutes and then resuspended in 0.9% saline before administration.

### Mice and treatments

Wild-type (WT) littermates and GPR41 knockout (*GPR41*^*-/-*^) mice on a C57BL/6J background were purchased from GemPharmatech (Nanjing, China). The animals were maintained under controlled condition (23–25℃) with a 12 hour light/dark cycle in the Animal Housing Unit of Jiangnan University (Jiangsu, China). Throughout the study, pups were housed with and nursed by the dam. The NEC was established in 10-day-old pups as described previously with appropriate adjustments.^18^ In brief, trinitrobenzene sulfonate (TNBS) at a dosage of 50 mg/kg were administered by gavage and rectal instillation. The *L. gasseri* FWJL-4 and LGG (ATCC 53,103) were administered by gavage at a dosage of 5 × 10^8^ CFU/mice daily for 4 days prior to TNBS treatment. To demonstrate that the protective role of *L. gasseri* in NEC is mediated by acetate, mice received *i.g*. treatment with 150 mM sodium acetate (SA, an exogenous acetate donor) daily for 4 days.^19^ The GPR43 inhibitor GPLG0974, was dissolved in 5% dimethyl sulfoxide in saline, as described previously, and administered intraperitoneally (1 mg/kg BW, *i.p*. once daily) prior to SA prophylaxis.^20,21^ Pups were monitored every 3 hours. At 24 hours post-treatment or upon the development of severe symptoms, the mice were anesthetized by 5% isoflurane inhalation, following by cardiac blood collection, removal of the small intestine, and collection of the ileum and ileal contents. Disease activity index (DAI), which evaluates weight loss, diarrheal condition, and hematochezia. The DAI was scored by the changes of weight loss (0, none; 1, 1 − 5%; 2, 6 − 10%; 3, 10–15%; 4, > 15%), diarrheal condition (0, normal; 2, soft; 4, diarrhea), and hematochezia (0, normal; 2, stool hemoccult positive; 4, bleeding around anus). The DAI scores were the average of the three indicators. During the experimental treatment, mice were observed at 0, 3, 6, 12, 24 h and the body weight changes, diarrheal condition, and diarrhea were recorded. After 24 hours, the mice were euthanized using 5% isoflurane inhalation followed by cervical dislocation, and the small intestine was removed for length measurement.

### Histopathological staining

Following abdominal incision, the gastrointestinal tract was carefully removed, and the terminal 2 cm of small intestine (ileum) were excised. The distal 0.5 cm of each fresh ileum were fixed in 4% paraformaldehyde overnight, washed with running water for 2 hours and then rehydrated through a graded ethanol series, embedded in paraffin, and sectioned into 4 μm slices by a Skiving Machine Slicer (Leica,Wetzlar, Germany). Periodic Acid-Schiff (PAS) staining was conducted according to the manufacturer’s instructions, and sections were stained with hematoxylin and eosin (H&E) using the standard procedure. Ileal morphology was visualized using a digital section scanner (Pannoramic, 3DHISTCH, Budapest, Hungary). Histopathological evaluation, following a scoring system adapted from previous studies: grade 0 = no injury; grade 1 = mild injury; disruption of villus tips or mild separation of lamina propria in ileum; no structural changes; grade 2 injury was considered moderate with midvillus disruption, clear separation of lamina propria, or submucosal edema; grade 3 = severe injury; severe submucosa separation or edema; grade 4 = transmural injury.^18,22^ The NEC score > 2 is considered to have NEC.

### Western blot

Ileal tissues were homogenized in ice-cold RIPA lysis buffer containing protease and phosphatase inhibitors. The supernatant was obtained by centrifugation (12,000 *g*, 4°C, 15 minutes), and protein concentration was determined using a Bicinchoninic acid (BCA) protein assay kit. Equal amounts of protein from each sample were separated by SDS-polyacrylamide gels and transferred onto nitrocellulose membranes. Membranes were blocked with skim milk for 1 hour, and incubated overnight at 4°C with primary antibodies. The following primary antibodies were used: Claudin-1 (Cat# 13050–1-AP, 1:1000, RRID: AB_2079881), ZO-1 (Cat# 21773-1AP, 1:1000, RRID: AB_10733242), Caspase-8 (Cat# 13423–1-AP, 1:500, RRID: AB_2068463), gasdermin D (GSDMD) (Cat# 66387–1-IG, 1:2000, RRID: AB_2881763) and mixed lineage kinase-like (MLKL) (Cat# 66675–1-IG, 1:5000, RRID: AB_2882029) from Proteintech (Hubei, China), Occludin (Cat# AP0765, 1:1000, RRID: AB_2797425 Biowrold, Jiangsu, China), p-MLKL (Cat# ab196436, 1:1000, RRID: AB_2687465, Abcam, CA, USA). Membranes were incubated with secondary antibodies conjugated to HRP for 2 hours. Protein expression was quantified by grayscale value analysis using ImageJ Software (NIH, USA) with Glyceraldehyde 3-phosphate dehydrogenase (GAPDH, Cat#AC002 1:10000, RRID: AB_2736879, Abclonal, Wuhan, China) as the internal control.

### Enzyme-linked immunosorbent assay (ELISA)

Blood was collected from pups by cardiac sampling, left for 30 minutes at room temperature, centrifuged at 3,000 *g* for 20 minutes, and serum was collected. Levels of serum C-reactive protein (CRP), serum amyloid A (SAA), and lipopolysaccharides (LPS) were measured using ELISA kits (MLBio, Shanghai, China) according to the manufacturer’s instructions.

### Immunofluorescence staining

Paraffin-embedded tissue sections were rehydrated serially in xylene and gradient ethanol, and antigen retrieval was performed using citrate buffer (Beyotime, Shanghai, China) 20 minutes. Sections were blocked with 10% goat serum for 1.5 hours and incubated overnight at 4°C with primary antibodies, including Mucin-2 (MUC2) (Cat# 27675–1-AP, 1:500, RRID: AB_2880943, Proteintech) and fatty acid binding protein 2 (FABP2) (Cat# 67691–1-IG, 1:200, RRID: AB_2882884, Proteintech). Secondary antibodies conjugated to fluorescein were applied for 1.5 hours, followed by nuclear staining with 4,6-diamino-2-phenyl indole (DAPI) for 10 minutes. Fluorescence images were captured using a Zeiss fluorescence microscope (Zeiss Axio Imager2, Oberkochen, Germany).

### Short-chain fatty acid (SCFA) analysis

SCFAs concentrations (acetate, propionate, and butyrate) in ileal contents and bacterial supernatant were analyzed by Gas Chromatography-Mass Spectrometer (GC-MS, Thermo Fisher Scientific, Waltham, USA) as previously described.^23^ Briefly, bacterial supernatant (1 mL) was deproteinated with 0.2 mL of 50% sulfuric acid, and diethyl ether (1 mL) was added to extract SCFAs. Samples were centrifuged at 18,000 *g* for 10 minutes, and supernatants were analyzed by GC-MS. For leal content analysis, 30–50 mg of ileal contents were firstly homogenized in 500 μL of saturated NaCl solution, acidified, and extracted as described above. SCFAs concentrations were quantified by Xcalibur software (Thermo Fisher Scientific) using the external standard method.

### Whole genome sequencing of L. gasseri FWJL-4

The *L. gasseri* FWJL-4 was cultured overnight in MRS medium, and the whole genome was sequenced at Sangon Biotech (Shanghai, China). Protein sequences were compared with the COG database using NCBI BLAST+ to obtain functional annotation information.

### Statistics analysis

All data are represented as mean ± standard deviation (SD). For survival rate statistical analysis, the log-rank test was used. Differences among three or more groups were determined using analysis of variance (ANOVA) followed by Tukey’s *post hoc* test. Two-way ANOVA was performed for body weight analysis. All data were analyzed using GraphPad Prism software (San Diego, USA).

### Role of funders

The funders have no role in study design, data collection, data analyses, interpretation, or writing of the report.

## Results

### L. gasseri FWJL-4 supplementation protects against NEC in mice

**Figure 1.**
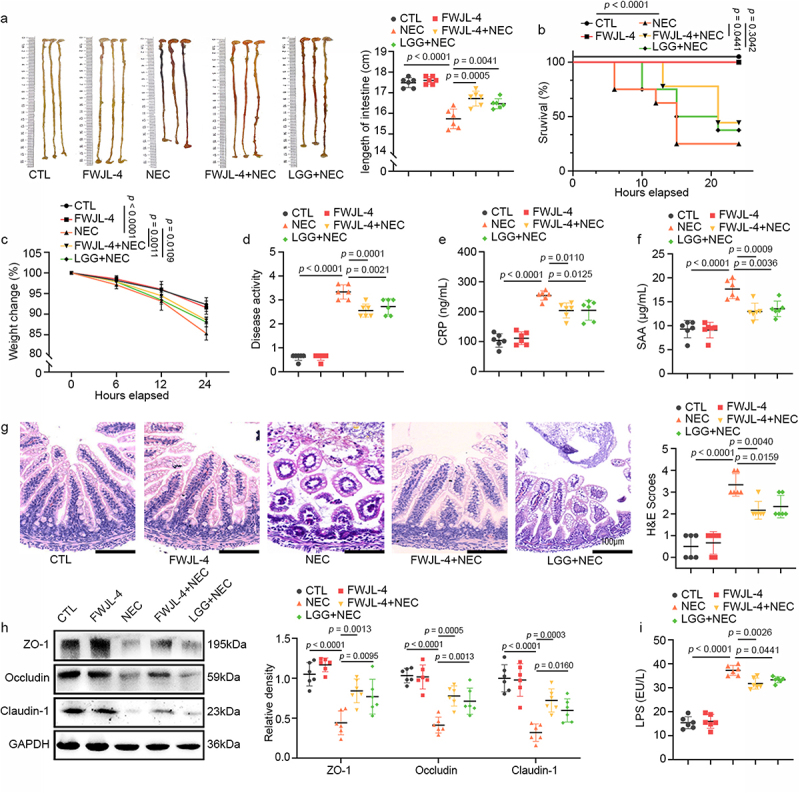
*L. gasseri* FWJL-4 or LGG (ATCC 53,103) supplementation alleviates NEC in mice. *L. gasseri* FWJL-4, LGG (ATCC 53,103, 5 × 10^8^ CFU/mouse), or normal saline were pre-gavaged for 4 days, followed by TNBS (50 mg/kg) to induce NEC in the mice. (a) Intestine length measured in mice subjected to NEC at 24 hours after *L. gasseri* FWJL-4 or LGG (ATCC 53,103) treatment, *n* = 6 per group. (b) Survival observed for 24 hours of mice subjected to NEC after *L. gasseri* FWJL-4 or LGG (ATCC 53,103) treatment, *n* = 8 per group. (c) Changes in body weight recorded in mice subjected to NEC at 24 hours after *L. gasseri* FWJL-4 or LGG (ATCC 53,103) treatment in relative to starting weight, *n* = 6 per group. (d) Disease activity index recorded during the mice subjected to NEC at 24 hours after *L. gasseri* FWJL-4 or LGG (ATCC 53,103) treatment according to weight change, hematochezia and diarrhea, *n* = 6 per group. (e) Serum CRP levels and (f) SAA levels measured in mice subjected to NEC at 24 hours after *L. gasseri* FWJL-4 or LGG (ATCC 53,103) treatment, *n* = 6 per group. (g) Representative photomicrographs show H&E-stained ileum and H&E scores of mice subjected to NEC at 24 hours after *L. gasseri* FWJL-4 or LGG (ATCC 53,103) treatment. Scale bar: 100 μm, *n* = 6 per group. (h) Western blot analysis and densitometry of TJPs (ZO-1, Occludin and claudin-1) measured in the ileum of mice subjected to NEC at 24 hours after *L. gasseri* FWJL-4 or LGG (ATCC 53,103) treatment, *n* = 6 per group. (i) Serum LPS levels measured in mice subjected to NEC at 24 hours after *L. gasseri* FWJL-4 or LGG (ATCC 53,103) treatment, *n* = 6 per group. Data are representative and were the mean ± SD from three independent experiments. *p* values were calculated by one-way ANOVA followed by Tukey’s *post hoc* test for multiple comparisons. For survival rate statistical analysis, data were expressed as Log-rank test, two-way ANOVA was performed for body weight analysis.

Administering *L. gasseri* FWJL-4 or LGG (ATCC 53,103), a positive control strain, for 4 days before mice subjected to NEC.^6,17^ As illustrated in Figures 1a–d, mice with NEC exhibited significant pathological features, including a shortened small intestine, elevated mortality rates, decreased body weight, and elevated disease activity index (DAI) scores. Intriguingly, the administration of *L. gasseri* FWJL-4 and LGG (ATCC 53,103) to NEC mice markedly alleviated these symptoms. Further assessment of the systemic inflammatory markers associated with NEC, such as serum C-reactive protein (CRP), and serum amyloid A, (SAA),^18^ revealed that *L. gasseri* FWJL-4 and LGG (ATCC 53,103) also significantly reduced the levels of these markers (Figure 1e,f). In addition, histological analysis of the ileum in NEC mice confirmed severe injury, characterized by disrupted villi, significant crypt loss, and a notable increase in histopathological scores. Importantly, treatment with *L. gasseri* FWJL-4 markedly improved these histological features (Figure 1g). The disruption of the gut barrier is regarded as a pathological hallmark of NEC.^1^ Western blot analysis demonstrated a substantial downregulation of tight junction proteins (TJPs), including Claudin-1, Occludin and ZO-1 in the ileum of NEC mice (Figure 1h). Remarkably, administration of *L. gasseri* FWJL-4 effectively upregulated their expression. Consistent with the increased expression of TJPs in the ileum, supplementation with *L. gasseri* FWJL-4 and LGG (ATCC 53,103) resulted in reduced intestinal permeability, as evidenced by a decrease in serum LPS levels (Figure 1i). Taken together, these data demonstrate that *L. gasseri* FWJL-4 provides significant protection against NEC.

### L. gasseri FWJL-4 increases the proportion of goblet cells and enterocytes in NEC mice

**Figure 2.**
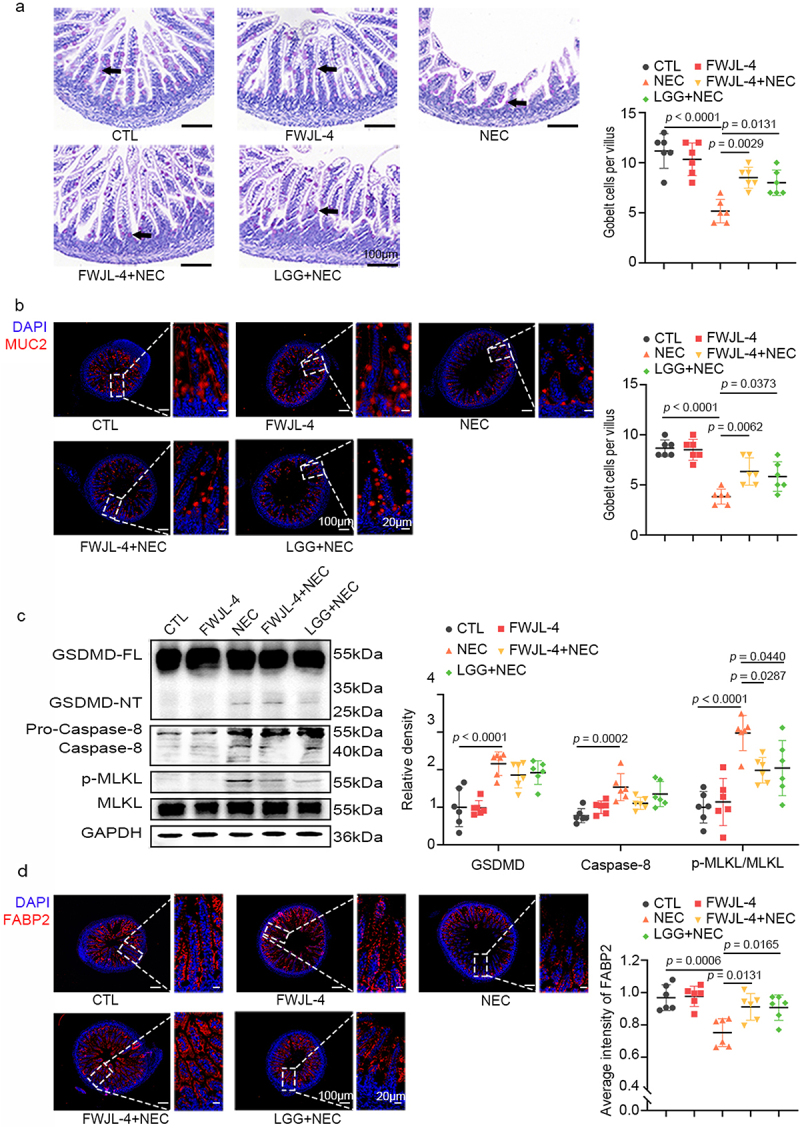
*L*. gasseri FWJL-4 or LGG (ATCC 53,103) enhances mucus production and epithelial cell integrity while regulating necroptosis in NEC mice. *L. gasseri* FWJL-4, LGG (ATCC 53,103, 5 × 10^8^ CFU/mouse), or normal saline were pre-gavaged for 4 days, followed by TNBS (50 mg/kg) to induce NEC in the mice. (a) Representative photomicrographs show PAS staining and the number of goblet cells per villus of mice subjected to NEC at 24 hours after *L. gasseri* FWJL-4 or LGG (ATCC 53,103) treatment. Goblet cells are indicated by arrows, *n* = 6 per group. (b) Representative photomicrographs show the localization and expression of MUC2 (red) in ileum of mice subjected to NEC at 24 hours after *L. gasseri* FWJL-4 or LGG (ATCC 53,103) treatment by immunofluorescent staining. Left, the panorama image, scale bar:100 μm; right, the magnified image, scale bar: 20 μm. Nuclei were stained with DAPI (blue), *n* = 6 per group. (c) Western blot analysis and densitometry of GSDMD, caspase-8, MLKL and p-mlkl measured in the ileum of mice subjected to NEC at 24 hours after *L. gasseri* FWJL-4 or LGG (ATCC 53,103) treatment, *n* = 6 per group. (d) Representative photomicrographs show the localization and expression of FABP2 (red) in ileum of mice subjected to NEC at 24 hours after *L. gasseri* FWJL-4 or LGG (ATCC 53,103) treatment by immunofluorescent staining. Left, the panorama image, scale bar:100 μm; right, the magnified image, scale bar: 20 μm. Nuclei were stained with DAPI (blue), *n* = 6 per group. Data are representative and were the mean ± SD from three independent experiments. *p* values were calculated by one-way ANOVA followed by Tukey’s *post hoc* test for multiple comparisons.

Ileal goblet cells, located within the mucosal epithelium, are crucial for secreting mucus that maintains intestinal barrier integrity.^24^ In NEC mice, PAS staining revealed a significant reduction in mucus levels, a decrease that was markedly attenuated by treatment with *L. gasseri* FWJL-4 or LGG (ATCC 53,103) (Figure 2a). Consistent with the PAS staining results, immunofluorescence staining revealed that treatment with *L. gasseri* FWJL-4 or LGG (ATCC 53,103) significantly enhanced MUC2 expression, which was otherwise downregulated in NEC mice (Figure 2b). Excessive intestinal epithelial cell death is a significant contributor to NEC development.^25^ We subsequently investigated the effect of *L. gasseri* FWJL-4 on the type of cell death in the ileum of NEC mice. GSDMD, Caspase-8, and MLKL phosphorylation serve as markers for pyroptosis, apoptosis, and necroptosis, respectively. Western blot analysis revealed increased levels of activated GSDMD, Caspase-8, and phosphorylated MLKL in NEC mice (Figure 2c). *L. gasseri* FWJL-4 significantly inhibited MLKL phosphorylation but did not affect the activation of GSDMD or Caspase-8, indicating that *L. gasseri* FWJL-4 specifically modulated necroptosis. We also examined whether *L. gasseri* FWJL-4 influenced the number of epithelial cells using FABP2 staining, a marker of epithelial cells. As anticipated, both *L. gasseri* and LGG (ATCC 53,103) treatment significantly increased the number of epithelial cells compared to NEC mice. (Figure 2d). Collectively, these results suggest that *L. gasseri* FWJL-4 exerts a protective intestinal barrier function by increasing number of goblet cells and enterocytes through the inhibition of necroptosis in the ileum.

### L. gasseri FWJL-4 ameliorates NEC through acetate production

**Figure 3.**
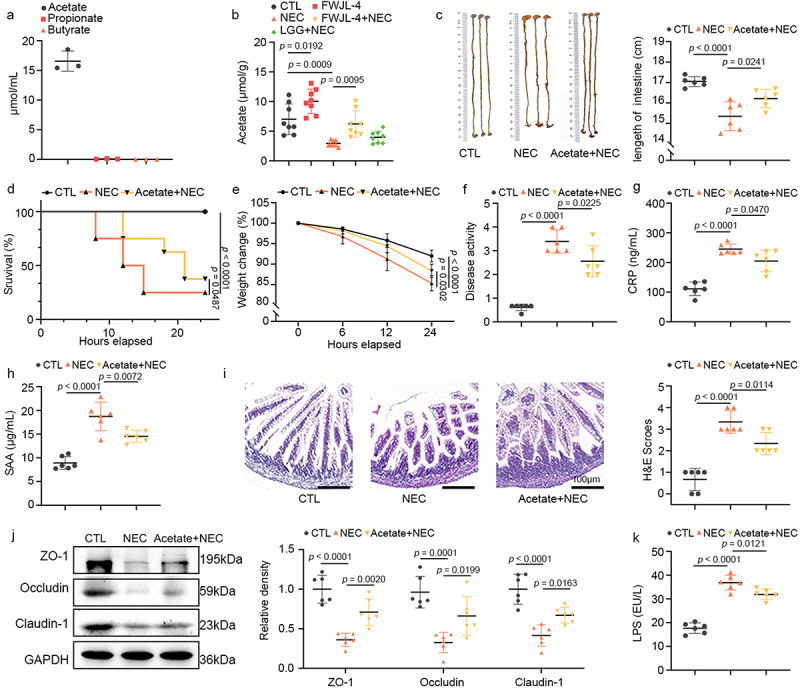
*L. gasseri* FWJL-4 produces acetate, and acetate supplementation alleviates NEC in mice. The levels of SCFAs (acetate, propionate and butyrate) in the supernatant of *L. gasseri* FWJL-4 and the ileal contents of mice were detected using GC-MS. Sodium acetate (SA, 150 mm/mouse), or normal saline were pre-gavaged for 4 days, followed by TNBS (50 mg/kg) to induce NEC in the mice. (a) Levels of acetate, propionate and butyrate measured in supernatant of *L. gasseri* FWJL-4, *n* = 3 per group. (b) Levels of acetate measured in ileal contents of mice subjected to NEC at 24 hours after *L. gasseri* FWJL-4 or LGG (ATCC 53,103) treatment, *n =* 8 per group. (c) Intestine length measured in mice subjected to NEC at 24 hours after acetate treatment, *n* = 6 per group. (d) Survival observed for 24 hours of mice subjected to NEC after acetate treatment, *n* = 8 per group. (e) Changes in body weight recorded in mice subjected to NEC at 24 hours after acetate treatment in relative to starting weight, *n* = 6 per group. (f) Disease activity index recorded during the mice subjected to NEC at 24 hours after acetate treatment according to weight change, hematochezia and diarrhea, *n* = 6 per group. (g) Serum CRP levels and (h) SAA levels measured in mice subjected to NEC at 24 hours after acetate treatment, *n* = 6 per group. (i) Representative photomicrographs show H&E-stained ileum and H&E scores of mice subjected to NEC at 24 hours after acetate treatment. Scale bar: 100 μm, *n* = 6 per group. (j) Western blot analysis and densitometry of TJPs (ZO-1, Occludin and claudin-1) measured in the ileum of mice subjected to NEC at 24 hours after acetate treatment, *n* = 6 per group. (k) Serum LPS levels measured in mice subjected to NEC at 24 hours after acetate treatment, *n* = 6 per group. Data are representative and were the mean ± SD from three independent experiments. *p* values were calculated by one-way ANOVA followed by Tukey’s *post hoc* test for multiple comparisons. For survival rate statistical analysis, data were expressed as Log-rank test, two-way ANOVA was performed for body weight analysis.

We next explored the molecular basis by which *L*. *gasseri* FWJL-4 confers protection against NEC. COG functional annotation of the *L. gasseri* FWJL-4 genome revealed a notable capacity for carbohydrate transport and metabolism, along with an abundance of functional genes related to acetate production (Figure S1a). Correspondingly, we detected significant levels of acetate in the supernatant of *L. gasseri* FWJL-4 (Figure 3a). Clinical studies have reported a significant reduction in fecal SCFAs, including acetate, propionate, and butyrate, in infants with NEC.^14^ Consistent with these findings, we observed a marked decrease in ileal SCFAs in NEC mice (Figure 3b, Figure S1b and c). Notably, *L. gasseri* FWJL-4 significantly increased the acetate levels in ileal contents of NEC mice, although it did not affect propionate or butyrate levels (Figure 3b, Figure S1b and c). To further verify acetate mediates the protective role of *L. gasseri* FWJL-4, we treated NEC mice with sodium acetate prior to TNBS administration. As shown in Figures 3c–h, exogenous acetate supplementation significantly attenuated small intestine shortening (Figure 3c), improved survival rates (Figure 3d), reduced weight loss (Figure 3e), decreased DAI scores (Figure 3f), and lowered serum CRP and SAA levels in NEC pups (Figure 3g,h). Moreover, acetate supplementation ameliorated ileal injury, villus disruption, and histopathological scores in NEC mice (Figure 3i). Western blot analysis further demonstrated that acetate treatment increased the expression of ileal TJPs (Claudin-1, Occludin and ZO-1), which were otherwise reduced in NEC mice (Figure 3j). Consistent with these results, acetate administration decreased intestinal permeability in NEC mice, as evidenced by reduction of serum LPS levels (Figure 3k). Collectively, our data suggest that *L. gasseri* FWJL-4 exerts its protective effects in NEC mice through the production of acetate.

### Acetate maintains goblet cell and enterocyte numbers in NEC mice

**Figure 4.**
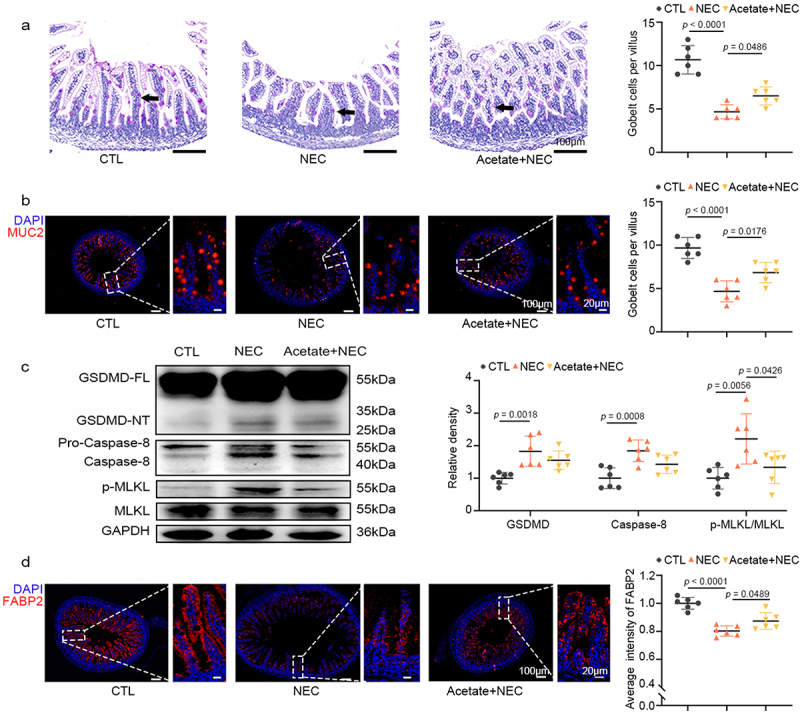
Acetate enhances mucus production and epithelial cell integrity while regulating necroptosis in NEC mice. SA (150 mM/mouse), or normal saline were pre-gavaged for 4 days, followed by TNBS (50 mg/kg) to induce NEC in the mice. (a) Representative photomicrographs show PAS staining and the number of goblet cells per villus of mice subjected to NEC at 24 hours after acetate treatment. Goblet cells are indicated by arrows, *n* = 6 per group. (b) Representative photomicrographs the localization and expression of MUC2 (red) in ileum of mice subjected to NEC at 24 hours after acetate treatment by immunofluorescent staining. Left, the panorama image, scale bar:100 μm; right, the magnified image, scale bar: 20 μm. Nuclei were stained with DAPI (blue), *n* = 6 per group. (c) Western blot analysis and densitometry of GSDMD, caspase-8, MLKL and p-mlkl measured in the ileum of mice subjected to NEC at 24 hours after acetate treatment, *n* = 6 per group. (d) Representative photomicrographs the localization and expression of FABP2 (red) in ileum of mice subjected to NEC at 24 hours after acetate treatment by immunofluorescent staining. Left, the panorama image, scale bar:100 μm; right, the magnified image, scale bar: 20 μm. Nuclei were stained with DAPI (blue), *n* = 6 per group. Data are representative and were the mean ± SD from three independent experiments. *p* values were calculated by one-way ANOVA followed by Tukey’s *post hoc* test for multiple comparisons.

To explore the effects of acetate, next we administered exogenous acetate to NEC mice. As expected, exogenous acetate supplementation significantly mitigated NEC-induced mucus loss in the ileum, as evidenced by PAS staining (Figure 4a), and increased MUC2 expression, as shown by immunofluorescent staining (Figure 4b). Consistent with the effects of *L. gasseri* FWJL-4 on cell death, acetate primarily inhibited MLKL phosphorylation, with no significant impact on the activation of GSDMD and Caspase 8 (Figure 4c). Additionally, acetate also upregulated the number of epithelial cells in NEC mice (Figure 4d). Taken together, these results suggest that *L. gasseri* FWJL-4 protects goblet cells and enterocytes and maintains mucus layer integrity by inhibiting necroptosis in the ileum through its metabolite, acetate.

### Blocking GPR43 or knocking out GPR41 abolishes the effects of acetate in NEC mice

**Figure 5.**
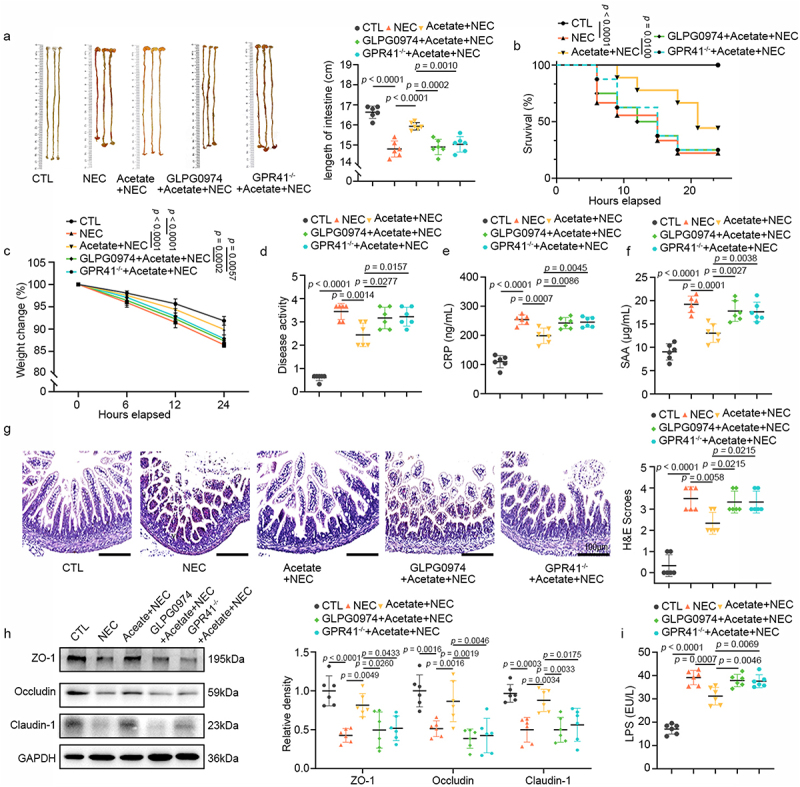
Blocking GPR43 or knocking out GPR41 abolishes the effects of acetate in NEC mice. *GPR41*^*-/-*^ mice or WT mice treated with GLPG0974 were pre-gavaged with SA (150 mM/mouse) for 4 days, followed by TNBS (50 mg/kg) to induce NEC in the mice. (a) intestine length measured in the WT mice treated with GPR43 antagonist and *GPR41*^*-/-*^ mice subjected to NEC at 24 hours after acetate treatment, *n* = 6 per group. (b) survival observed for 24 hours of the WT mice treated with GPR43 antagonist and *GPR41*^*-/-*^ mice subjected to NEC after acetate treatment, *n* = 8 per group. (c) Changes in body weight recorded in the WT mice treated with GPR43 antagonist and *GPR41*^*-/-*^ mice subjected to NEC at 24 hours after acetate treatment in relative to starting weight, *n* = 6 per group. (d) disease activity index recorded during the WT mice treated with GPR43 antagonist and *GPR41*^*-/-*^ mice subjected to NEC at 24 hours after acetate treatment according to weight change, hematochezia and diarrhea, *n* = 6 per group. (e) Serum CRP levels and (f) SAA levels measured in the WT mice treated with GPR43 antagonist and *GPR41*^*-/-*^ mice subjected to NEC at 24 hours after acetate treatment, *n* = 6 per group. (g) Representative photomicrographs show H&E-stained ileum and H&E scores of the WT mice treated with GPR43 antagonist and *GPR41*^*-/-*^ mice subjected to NEC at 24 hours after acetate treatment. Scale bar: 100 μm, *n* = 6 per group. (h) Western blot analysis and densitometry of TJPs (ZO-1, Occludin and claudin-1) measured in the ileum of the WT mice treated with GPR43 antagonist and *GPR41*^*-/-*^mice subjected to NEC at 24 hours after acetate treatment, *n* = 6 per group. (i) Serum LPS levels measured in the WT mice treated with GPR43 antagonist and *GPR41*^*-/-*^ mice subjected to NEC at 24 hours after acetate treatment, *n* = 6 per group. Data are representative and were the mean ± SD from three independent experiments. *p* values were calculated by one-way ANOVA followed by Tukey’s *post hoc* test for multiple comparisons. For survival rate statistical analysis, data were expressed as Log-rank test, two-way ANOVA was performed for body weight analysis.

**Figure 6.**
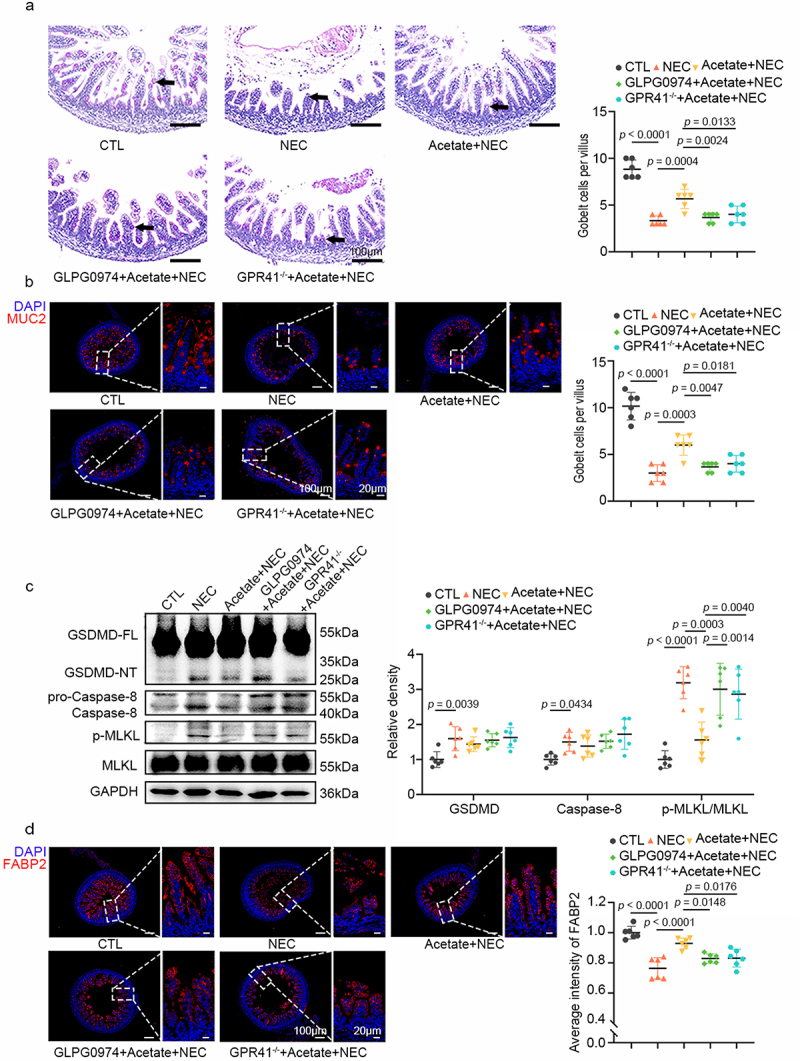
GPR43 and GPR41 mediate the effects of acetate in preserving goblet cells, enterocytes, and in mitigating necroptosis in NEC mice. *GPR41*^*-/-*^ mice or WT mice treated with GLPG0974 were pre-gavaged with SA (150 mM/mouse) for 4 days, followed by TNBS (50 mg/kg) to induce NEC in the mice. (a) Representative photomicrographs show PAS staining and the number of goblet cells per villus of the WT mice treated with GPR43 antagonist and *GPR41*^*-/-*^ mice subjected to NEC at 24 hours after acetate treatment. Goblet cells are indicated by arrows, *n* = 6 per group. (b) Representative photomicrographs the localization and expression of MUC2 (red) in ileum of the WT mice treated with GPR43 antagonist and *GPR41*^*-/-*^ mice subjected to NEC at 24 hours after acetate treatment by immunofluorescent staining. Left, the panorama image, scale bar:100 μm; right, the magnified image, scale bar: 20 μm. Nuclei were stained with DAPI (blue), *n* = 6 per group. (c) Western blot analysis and densitometry of GSDMD, caspase-8, MLKL and p-MLKL measured in the ileum of the WT mice treated with GPR43 antagonist and *GPR41*^*-/-*^ mice subjected to NEC at 24 hours after acetate treatment, *n* = 6 per group. (d) Representative photomicrographs the localization and expression of FABP2 (red) in ileum of the WT mice treated with GPR43 antagonist and *GPR41*^*-/-*^ mice subjected to NEC at 24 hours after acetate treatment by immunofluorescent staining. Left, the panorama image, scale bar:100 μm; right, the magnified image, scale bar: 20 μm. Nuclei were stained with DAPI (blue), *n* = 6 per group. Data are representative and were the mean ± SD from three independent experiments. *p* values were calculated by one-way ANOVA followed by Tukey’s *post hoc* test for multiple comparisons.

GPR43 and GPR41 are receptors for SCFAs, with acetate serving as a primary agonist. These receptors are predominantly expressed in the gut and play a crucial role in regulating various physiological functions.^21,26^ To further elucidate the receptor involvement in the protective effects of acetate against NEC, we employed the GPR43 inhibitor GLPG0974 or GPR41 knockout (*GPR41*^*-/-*^) mice. These models were used to determine whether the beneficial effects of *L. gasseri* FWJL-4-derived acetate are mediated through GPR43 or GPR41. As shown in Figures 5a–f, the preventive efficacy of acetate against NEC was compromised in both WT mice treated with GLPG0974 and *GPR41*^*-/-*^ mice. Furthermore, the protective influence of acetate on gut barrier function was abolished upon inhibition of GPR43 or knockout of GPR41, as evidenced by increased ileal histopathological scores (Figure 5g), decreased expression of ileal TJP (Figure 5h), and elevated serum LPS levels (Figure 5i). Additionally, acetate supplementation significantly mitigated NEC-induced goblet cell loss and increased MUC2 expression, effects that were blocked by GLPG0974 treatment or GPR41 knockout (Figures 6a,b). Consistently, the inhibition of GPR43 or knockout of GPR41 reversed the protective effect of acetate against ileal necroptosis (Figure 6c). Finally, the significant increase in enterocytes number mediated by exogenous acetate supplementation was also thwarted in both *GPR41*^−/−^ mice and WT mice treated with GLPG0974 (Figure 6d). Taken together, these data suggest that the protective effects of acetate against NEC are depended on GPR43 and GPR41.

### L. gasseri FWJL-4-mediated protective effect on NEC is dependent on GPR43 and GPR41

**Figure 7.**
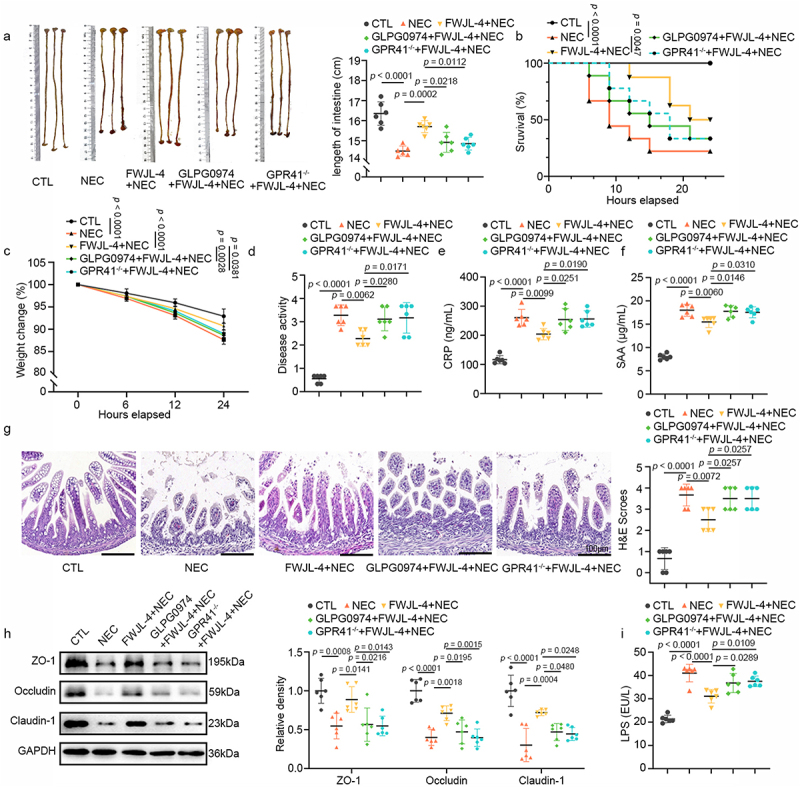
*L. gasseri* FWJL-4-mediated protective effect on NEC is dependent on GPR43 and GPR41. *GPR41*^*-/-*^ mice or WT mice treated with GLPG0974 were pre-gavaged with *L. gasseri* FWJL-4 (5 × 10^8^ CFU/mouse) for 4 days, followed by TNBS (50 mg/kg) to induce NEC in the mice. (a) Intestine length measured in the WT mice treated with GPR43 antagonist and *GPR41*^*-/-*^ mice subjected to NEC at 24 hours after *L. gasseri* FWJL-4 treatment, *n* = 6 per group. (b) Survival observed for 24 hours of the WT mice treated with GPR43 antagonist and *GPR41*^*-/-*^ mice subjected to NEC after *L. gasseri* FWJL-4 treatment, *n* = 8 per group. (c) Changes in body weight recorded the WT mice treated with GPR43 antagonist and *GPR41*^*-/-*^ mice subjected to NEC at 24 hours after *L. gasseri* FWJL-4 treatment in relative to starting weight, *n* = 6 per group. (d) Disease activity index recorded during the WT mice treated with GPR43 antagonist and *GPR41*^*-/-*^ mice subjected to NEC at 24 hours after *L. gasseri* FWJL-4 treatment according to weight change, hematochezia and diarrhea, *n* = 6 per group. (e) Serum CRP levels and (f) SAA levels measured in the WT mice treated with GPR43 antagonist and *GPR41*^*-/-*^ mice subjected to NEC at 24 hours after *L. gasseri* FWJL-4 treatment, *n* = 6 per group. (g) Representative photomicrographs show H&E-stained ileum and H&E scores of the WT mice treated with GPR43 antagonist and *GPR41*^*-/-*^ mice subjected to NEC at 24 hours after *L. gasseri* FWJL-4 treatment. Scale bar: 100 μm, *n* = 6 per group. (h) Western blot analysis and densitometry of TJPs (ZO-1, Occludin and claudin-1) measured in the ileum of the WT mice treated with GPR43 antagonist and *GPR41*^*-/-*^mice subjected to NEC at 24 hours after *L. gasseri* FWJL-4 treatment, *n* = 6 per group. (i) Serum LPS levels measured in the WT mice treated with GPR43 antagonist and *GPR41*^*-/-*^ mice subjected to NEC at 24 hours after *L. gasseri* FWJL-4 treatment, *n* = 6 per group. Data are representative and were the mean ± SD from three independent experiments. *p* values were calculated by one-way ANOVA followed by Tukey’s *post hoc* test for multiple comparisons. For survival rate statistical analysis, data were expressed as Log-rank test, two-way ANOVA was performed for body weight analysis.

**Figure 8.**
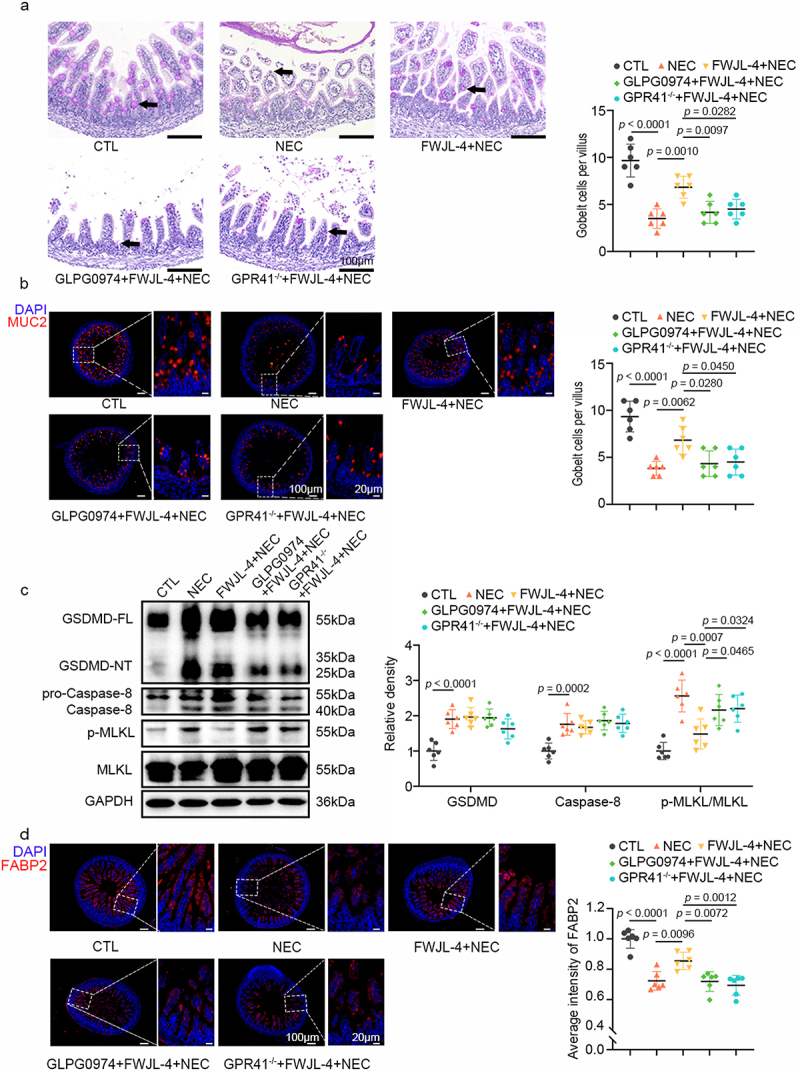
GPR43 and GPR41 modulate the protective effects of *L. gasseri* FWJL-4 against NEC. *GPR41*^*-/-*^ mice or WT mice treated with GLPG0974 were pre-gavaged with *L. gasseri* FWJL-4 (5 × 10^8^ CFU/mouse) for 4 days, followed by TNBS (50 mg/kg) to induce NEC in the mice. (a) Representative photomicrographs show PAS staining and the number of goblet cells per villus of the WT mice treated with GPR43 antagonist and *GPR41*^*-/-*^ mice subjected to NEC at 24 hours after *L. gasseri* FWJL-4 treatment. Goblet cells are indicated by arrows, *n* = 6 per group. (b) Representative photomicrographs the localization and expression of MUC2 (red) in ileum of the WT mice treated with GPR43 antagonist and *GPR41*^*-/-*^ mice subjected to NEC at 24 hours after *L. gasseri* FWJL-4 treatment by immunofluorescent staining. Left, the panorama image, scale bar:100 μm; right, the magnified image, scale bar: 20 μm. Nuclei were stained with DAPI (blue), *n* = 6 per group. (c) Western blot analysis and densitometry of GSDMD, caspase-8, MLKL and p-MLKL measured in the ileum of the WT mice treated with GPR43 antagonist and *GPR41*^*-/-*^ mice subjected to NEC at 24 hours after *L. gasseri* FWJL-4 treatment, *n* = 6 per group. (d) Representative photomicrographs the localization and expression of FABP2 (red) in ileum of the WT mice treated with GPR43 antagonist and *GPR41*^*-/-*^ mice subjected to NEC at 24 hours after *L. gasseri* FWJL-4 treatment by immunofluorescent staining. Left, the panorama image, scale bar:100 μm; right, the magnified image, scale bar: 20 μm. Nuclei were stained with DAPI (blue), *n* = 6 per group. Data are representative and were the mean ± SD from three independent experiments. *p* values were calculated by one-way ANOVA followed by Tukey’s *post hoc* test for multiple comparisons.

To further confirm that the protective effects of *L. gasseri* FWJL-4-derived acetate on NEC were mediated through GPR43 and GPR41, we administered *L. gasseri* FWJL-4 to *GPR41*^−/−^ mice or WT mice treated with GLPG0974. Consistent with the results observed with acetate treatment in these models, both GLPG0974 administration and GPR41 knockout abolished the protective effects of *L. gasseri* FWJL-4 on NEC, as evidenced by reduced small intestine length (Figure 7a), decreased survival rates (Figure 7b), weight loss (Figure 7c), increased DAI (Figure 7d), and elevated serum CRP and SAA levels (Figure 7e,f). Moreover, we observed an increase in the histopathological scores (Figure 7g), a decrease in the expression of ileal TJPs (Figure 7h), and an increase in serum LPS levels (Figure 7i) following GLPG0974 treatment or GPR41 knockout, compared to *L. gasseri* FWJL-4 treated NEC WT mice. These findings indicated GLPG0974 treatment or GPR41 knockout mitigated the protective effects of *L. gasseri* FWJL-4 on NEC-induced gut barrier dysfunction. Additionally, supplementation with *L. gasseri* FWJL-4 significantly reduced NEC-mediated goblet cell loss and increased MUC2 expression, effects that were also reversed by GLPG0974 treatment or GPR41 knockout (Figure 8a,b). Similarly, GLPG0974 or GPR41 knockout attenuated the effects of *L. gasseri* FWJL-4 on NEC-mediated ileal necroptosis (Figure 8c). Finally, in WT mice treated with GLPG0974 and *GPR41*^−/−^ mice, the significant increase in enterocyte number mediated by *L. gasseri* FWJL-4 supplementation was also thwarted (Figure 8d). Taken together, these data indicate that the protective effects of *L. gasseri* FWJL-4 on NEC *via* acetate are dependant on GPR43 and GPR41.

## Discussion

In this investigation, we discovered that *L. gasseri* FWJL-4 enhanced intestinal barrier function and exhibited protective effects against experimental NEC in mice. Notably, *L. gasseri* FWJL-4 significantly increased acetate levels in the ileal content of NEC mice. Mechanistically, we further revealed that *L. gasseri* FWJL-4-derived acetate maintains the number of enterocytes and goblet cells and inhibits ileal necroptosis in a GPR43- and GPR41-dependent manner, thereby enhancing gut barrier function and attenuating NEC in mice.

The etiology of NEC is still unclear, but recent studies have identified intestinal microbiota disruption as an early hallmark of the disease.^27^ A critical factor in neonatal gut colonization, especially in the context of NEC, is the feeding method.^5,28^ Research has demonstrated that different feeding practices significant influence the development of an infant’s gut microbiota and overall health. Breastfeeding, rich in probiotics such as *Bifidobacteria* and *Lactobacilli*, promotes a balanced microbial environment and supports gut health.^29^ In contrast, the gut microbiota of NEC patients often shows an increased abundance of pathogens, such as *Enterococcus faecalis*, *Clostridioides difficile*, *Pseudomonas aeruginosa*, and members of the *Klebsiella* genus.^30^ Furthermore, infants with NEC are frequently unable to receive breast milk, which exacerbates gut microbiota disruption, leading to a microbiota dominated by *Streptococci* and *Staphylococci*.^29^ However, supplementation of breast milk with probiotics or prebiotics can help regulate neonatal intestinal colonization by reducing the growth of pathogenic microorganisms, increasing the presence of beneficial microbial species such as *Actinobacteria* and *Firmicutes*, and promoting the production of SCFAs.^31^ SCFAs like acetate, propionate and butyrate are crucial metabolites for maintaining intestinal homeostasis. They serve as essential fuels for intestinal epithelial cells and play a critical role in enhancing intestinal barrier function and immune regulation.^32^ Thus, SCFAs and SCFAs-producing bacteria may be potential targets for the protection and treatment of NEC.^33^

This study employed the method developed by Mohan Kumar et al., which employed TNBS to induce NEC in 10-day-old mice *via* gavage and rectal administration.^18^ The study compared the activated transcriptional networks in children with NEC to those in TNBS-induced NEC in mice. Specifically, TNBS-induced intestinal injury in mice closely mirrored human NEC in terms of cellular inflammatory responses and the expression of cytokines and chemokines in the affected intestine.^22,34^ In the study, H&E images demonstrated similar pathological processes, including villus destruction, loss, shedding, and necrosis (Figure 1g). Additionally, levels of CRP and SAA in the serum of NEC mice were elevated (Figure 1e, f).

NEC is characterized by intestinal ischemia and inflammation, which lead to the disruption of the intestinal epithelial barrier.^28^ The barrier disruption allows abnormal bacterial translocation, exacerbating the inflammatory response and worsening intestinal injury.^35^ *L. gasseri* has demonstrated the capacity to enhance intestinal barrier function and restore microbiota homeostasis, offering a potential strategy for protecting against NEC.^8,36^ In patients with NEC, there is a reduction in the abundance of protective or health-promoting bacteria, such as *L. gasseri*.^7^ Therefore, supplementing with *L. gasseri* may be a crucial intervention for restoring intestinal epithelial barrier in affected infants. Consistent with previous findings,^37^ our study confirms that the treatment with *L. gasseri* FWJL-4, a novel probiotic isolated from infant feces, upregulates TJPs and MUC2 expression, thereby reducing the severity of NEC. These results suggest that *L. gasseri* FWJL-4 represents a promising new probiotic option for the treatment of NEC. Although *L. gasseri* has demonstrated promising protective effects in animal models of NEC, its direct efficacy in clinical settings, especially for infants with NEC, has not been validated through large-scale human trials. Therefore, while preliminary animal research results are encouraging, further clinical studies are necessary to confirm the safety and efficacy of this strain for infants with NEC. The present study will enhance our understanding of its potential mechanisms and provide a scientific foundation for future therapeutic applications.

Acetate, while easier to produce, store, and administer than live probiotics, lacks the symbiotic benefits of *L. gasseri*, which can colonize the neonatal gut.^38^ This colonization enables continuous and regulated release of acetate without repeated administration – a significant advantage for neonates. Additionally, *L. gasseri* interacts with the host gut microbiome, regulates the immune system, and enhances intestinal barrier function, and maintains gut health by establishing a dynamic balance with other gut microorganisms.^39^ As a member of the lactic acid bacteria, *L. gasseri* produces various active metabolites, including lactic acid, bacteriocins, and short-chain fatty acids (SCFAs), which help it thrive in competitive microbial environments.^40^ Notably, *L. gasseri* is known for its production of antimicrobial peptides and other bioactive substances, including gassericins and SCFAs like acetate, which plays a pivotal role in maintaining intestinal barrier integrity.^16,41^ *In vitro* studies have demonstrated that acetate increases MUC2 levels in T84 cells,^42^ while *in vivo* experiments reveal its ability to lower ileal pH, reducing the presence of pathogenic microbes.^43^ Additionally, acetate exhibits anti-inflammatory properties by inhibiting the expression of pro-inflammatory factors such as IL-1α and TNF-α through the activation of GPR43.^44^ Acetate also inhibits the ERK/JNK/NF-κB pathway by increasing GPR41 expression.^45^ Importantly, our study highlights the crucial role of acetate in mediating the protective effects of *L. gasseri* FWJL-4 against NEC. Necroptosis of intestinal epithelial cells can directly damage the integrity of mucosal barrier and amplify the inflammatory response, further exacerbating NEC.^46^ A key finding in our study was that *L. gasseri* FWJL-4 inhibited the phosphorylation of MLKL, preventing ileal necroptosis and protecting goblet cells and enterocytes. In response to the necroptosis inhibition, *L. gasseri* FWJL-4 reduced ileal injury and inflammation. Using GPR41 knockout mice or a specific GPR43 inhibitor, we illustrated that acetate secreted by *L*. *gasseri* FWJL-4 activates the GPR43 or GPR41 in the ileum, contributing to its regulatory role on TJPs, MUC2, and the MLKL pathway. These findings underscore the significance of *L*. *gasseri* FWJL-4 in NEC treatment.

In conclusion, our investigation provides valuable insights into the protective mechanisms of *L. gasseri* FWJL-4 in mitigating NEC. The enhancement of intestinal barrier function and the inhibition of necroptosis by *L. gasseri* FWJL-4, mediated through acetate secretion and dependent on GPR43 and GPR41, represent a promising preventive intervention for NEC.

## Supplementary Material

Supplementarymaterial clean.docx

## Data Availability

The data that support the findings of this study will be available in Mendeley Data at DOI: 10.17632/h8mzyzfmt9.1.

## Data sharing statement

All primary data in this study will be made available upon request to the corresponding authors for individuals with appropriate data sharing agreements in place.

## List of abbreviations

Analysis of variance: ANOVA
American Type Culture Collection: ATCC
Bicinchoninic acid: BCA
Colony-forming units: CFU
C-reactive protein: CRP
4,6-diamino-2-phenyl indole: DAPI
Disease activity index: DAI
Enzyme-linked Immunosorbent Assay: ELISA
Fatty acid binding protein 2: FABP2
Guangdong Microbial Culture Collection Center: GDMCC
G protein-coupled receptor: GPR
GPR41 knockout: *GPR41*^*-/-*^
Glyceraldenhyde 3-phosphate dehtdrogenase: GAPDH
Gas Chromatography-Mass Spectrometer: GC-MS
Gasdermin D: GSDMD
Hematoxylin and eosin: H&E
*Lactobacillus gasseri*: *L. gasseri*
*Lactobacillus rhamnosus* GG: LGG
Lipopolysaccharides: LPS
Mixed lineage kinase-like: MLKL
deMan-Rogosa-Sharpe: MRS
Mucin-2: MUC2
Necrotizing enterocolitis: NEC
Periodic Acid-Schiff: PAS
Sodium acetate: SA
Serum amyloid A: SAA
Short-chain fatty acids: SCFAs
Standard deviation: SD
Trinitrobenzene sulfonate: TNBS
Tight junction proteins: TJPs
Wild-type: WT
