# Supplementary material for: Infant feces-derived Lactobacillus gasseri FWJL-4 mitigates experimental necrotizing enterocolitis via acetate production
Source: Gut Microbes. 2024 Dec 8;16(1):2430541. doi: 10.1080/19490976.2024.2430541 (PMC11633162; doi:10.1080/19490976.2024.2430541)
Supplement: Supplementarymaterial clean.docx [file KGMI_A_2430541_SM0463.docx]

Supplementary material for

Infant feces-derived *Lactobacillus gasseri* FWJL-4 mitigates experimental necrotizing enterocolitis via acetate production


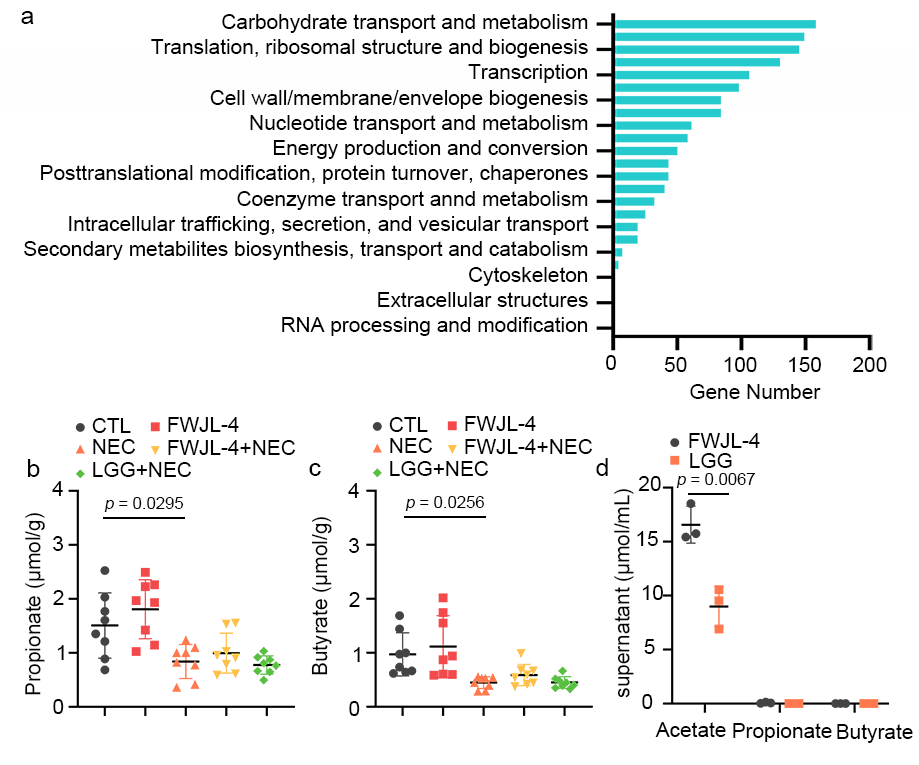


Figure S1, related to Figure 3: *L. gasseri* FWJL-4 produce acetate, and administering acetate 4 days before mice subjected to NEC can also prevent the disease.

(a) Genome-wide COG prediction of *L. gasseri* FWJL-4.

(b) Levels of propionate measured in ileal contents of mice subjected to NEC at 24 hours after *L. gasseri* FWJL-4 or LGG (ATCC 53103) treatment, *n =* 8 per group.

(c) Levels of butyrate measured in ileal contents of mice subjected to NEC at 24 hours after *L. gasseri* FWJL-4 or LGG (ATCC 53103) treatment, *n =* 8 per group.

(d) Levels of acetate, propionate and butyrate measured in supernatant of *L. gasseri* FWJL-4 and LGG (ATCC 53103), *n* = 3 per group.

Data are representative and were the mean SD from three independent experiments. *P* values were calculated by one-way ANOVA followed by Tukey’s *post hoc* test for multiple comparisons.
